# Impact of ascorbic acid in reducing the incidence of vancomycin associated nephrotoxicity in critically ill patients: A preliminary randomized controlled trial

**DOI:** 10.12688/f1000research.55619.1

**Published:** 2021-09-16

**Authors:** Nouran Hesham El-Sherazy, Naglaa Samir Bazan, Sara Mahmoud Shaheen, Nagwa A. Sabri

**Affiliations:** 1 Critical Care Medicine Department, Cairo University Hospitals, Cairo University, 11562, Egypt; 2 Clinical Pharmacy Department, Ain Shams University, Cairo, 11566, Egypt

**Keywords:** vancomycin, nephrotoxicity, critically ill patients, ascorbic acid

## Abstract

**Background** Antioxidants show nephroprotective effect against vancomycin associated nephrotoxicity (VAN) in animals. This study aimed to assess the ascorbic acid nephro-protective role against VAN clinically.

**Methods** Forty-one critically ill patients were randomly assigned to one of two groups: intervention group (vancomycin IV plus ascorbic acid, n=21) or control group (vancomycin IV only, n=20). Primary outcomes were the incidence of VAN and the absolute change in creatinine parameters, while mortality rate was the secondary outcome. Nephrotoxicity was defined as an increase in serum creatinine (S.cr) by at least 0.5 mg/dL or 50% of baseline for at least two successive measurements. This study is registered at Clinicaltrials.gov (NCT03921099), April 2019.

**Results** Mean absolute S.cr increase was significant when compared between both groups, *P*-value = 0.036, where S.cr increased by 0.05(0.12) and 0.34(0.55) mg/dL in the intervention and control groups, respectively. Mean absolute Cr.cl decline was significant when compared between both groups, *P*-value = 0.04, where Cr.cl was decreased by 5.9(17.8) and 22.3(30.4) ml/min in the intervention and control groups, respectively. Incidence of VAN was 1/21(4.7%) versus 5/20(25%) in the intervention and control groups, respectively (RR: 0.19; CI: 0.024–1.49; *P*-value = 0.093). Mortality was higher in the control group; however, it was not statistically significant, *P-*value = 0.141.

**Conclusion** Co-administration of ascorbic acid with vancomycin preserved renal function and reduced the absolute risk of VAN by 20.3%, however, the reduction in VAN incidence didn’t reach statistical significance level. Further large multicenter prospective trials are recommended.

## Introduction

Vancomycin (a glycopeptide antibiotic) has been the first line of treatment for methicillin-resistant *Staphylococcus aureus* (MRSA), methicillin-resistant coagulase-negative *Staphylococci* and *Enterococcus faecium* since 1970. ^1^ Over the past decades, the use of vancomycin has intensely increased due to the increased incidence of both the community and the health care MRSA infections. ^2^ Moreover, it has been intensively used for the sake of appropriate empirical coverage. ^3^

Trough level between 15–20 mg/L was recommended in complicated infections caused by *Staphylococcus aureus* as a surrogate marker for the pharmacokinetic/pharmacodynamic ratio (AUC/MIC more than 400). ^4^ However recent guidelines recommend area under the curve (AUC) monitoring using Bayesian software programs instead of trough level as the most accurate way for vancomycin dosing and monitoring. ^5^ In patients suffering serious MRSA infections, an individualized target of the AUC/MIC should be 400 to 600 (assuming MIC= 1 mg/L) in order to achieve clinical efficacy and improve patient safety. ^5^ Doses of 15 to 20 mg/kg (based on actual body weight every 8 to 12 hours) are usually recommended for most patients with normal renal function. ^5^

The incidence of vancomycin associated nephrotoxicity (VAN) is high and may reach 10–20% upon administering the conventional dose of vancomycin (one gram every 12 hours) and 30–40% upon administering the high dose (15–20 mg/kg every 8–12 hours). ^6^ It usually occurs within 4–8 days after initiating the therapy ^7^ and it is usually reversible within seven days in 44–75% of the patients upon discontinuing the treatment. ^7^ In addition, VAN can be reversed by adjusting doses correctly after renal impairment. ^8^ Some preventive strategies are used such as adequate hydration and therapeutic drug monitoring in high-risk patients ^9^; however, the incidence is still high, especially among critically ill patients.

Many risk factors have been found to increase the risk of VAN such as long duration of treatment (> seven days), ^7^ critical illness, ^7^ obesity (>101.4 kg), ^10, 11^ method of administration (intermittent infusion > continuous infusion), ^12, 13^ higher trough levels > 20 mg/L ^14^ and the use of two or more concomitant nephrotoxins [e.g. aminoglycosides, angiotensin converting enzyme inhibitors (ACEIs), non-steroidal anti-inflammatory drugs (NSAIDs), furosemide, cyclosporins, amphotericin B, and cisplatin]. ^10, 15^ Co-administrating piperacillin/tazobactam antibiotic with vancomycin was noted to be a possible risk that may enhance the incidence of VAN compared to administering vancomycin alone in 2011. Then subsequent studies were done but the results are still conflicting. ^16– 18^ Also, flucloxacillin has been reported recently to increase the risk for acute kidney injury (AKI) in patients receiving vancomycin. ^19^

Oxidative stress has been thought to be the main cause of nephrotoxicity associated with vancomycin. The reactive oxygen species (ROS) generated by the mitochondria initiate renal cell apoptosis, which results in renal dysfunction. ^7^ These generated ROS decrease the activity of superoxide dismutase and catalase enzymes, which have a defensive antioxidative role. ^7^

The belief that oxidative stress plays a role in renal dysfunction has led to the concept that antioxidants can be beneficial in a nephro-protection approach. Ascorbic acid is an antioxidant that acts as a free radical scavenger, therefore it can reduce renal stress. ^20^ It has been shown to be nephro-protective in animals against the stress generated after cardiac ischemia and by medications (e.g. gentamicin, vancomycin, cisplatin and colistimethate). ^7, 21^ With regard to safety, it has been reported to be the least toxic of all vitamins. Generally, it is well tolerated by patients while some may experience minimal gastrointestinal side effects. ^22^ Moreover, it’s cheap and readily available on the market.

At the moment, no randomized controlled clinical trial has been carried out to investigate the role of ascorbic acid in preventing VAN. Therefore, this pilot study aims to investigate the possible nephro-protective effect of ascorbic acid against VAN in critically ill patients.

## Methods

### Ethics

The study was performed to comply with the Declaration of Helsinki principles. ^23^ Approvals were obtained from the Ethics Committee and institutional review board of the Faculty of Pharmacy, Ain Shams University (No: 208) on 10 ^th^ September 2018, and the council of the critical care medicine department at Cairo University Hospitals. The study is registered at Clinicaltrials.gov (Registration: NCT03921099), April 2019. A written informed consent was obtained from each participating patient or from his/her next first relative.

### Setting

This was an open-label prospective, randomized, controlled study conducted on critically ill patients. Patients were recruited from the critical care medicine department at Cairo University Hospitals, Cairo, Egypt, during the period between April 2019 and December 2019.

### Patients

All patients suffering from clinical signs of infection in the critical care medicine department were screened for the following inclusion criteria: adults (age >18) of both genders with susceptibility to gram-positive infection (MRSA) that required vancomycin treatment for at least 72 hours. Patients were excluded if they had a known allergy to either vancomycin or ascorbic acid, pregnant or lactating females, baseline serum creatinine ≥2mg/dL, receiving other nephrotoxic drugs (e.g. aminoglycosides, amphotericin B, cisplatin, colistimethate sodium or calcinurine inhibitors) at time of initiating vancomycin, suffer from glucose-6-phosphate dehydrogenase deficiency or urinary tract stones, or were expected to undergo contrast medium administration within seven days.

### Randomization

Eligible patients were assigned to either the vancomycin-ascorbic acid (intervention) group or the vancomycin (control) group by block randomization using an online program for randomization ( http://www.randomizer.org). Patients were subjected to randomization using sequentially numbered opaque sealed envelopes (SNOSE), to accomplish allocation concealment. ^24^

Vancomycin was administered intravenously at a dose of 15–20 mg/kg based on actual body weight every 8–12 hours in both groups. Vancomycin powder was reconstituted by adding 0.9% normal saline (NS) to a final concentration of 2.5–5 mg/ml. ^5^ Reconstituted powder was infused slowly with a maximum rate of 10 mg/min over at least one hour (infusion time was increased to 1.5–2 hours if the patient’s single dose was greater than one gram to avoid Red man syndrome). ^5^

Ascorbic acid was administered orally at a dose of two grams twice daily half an hour before the vancomycin dose for the patients assigned to the intervention group. ^25^ No hazardous effects were expected from either administering the ascorbic acid dose or in combination with vancomycin in the vancomycin regimen. For those patients in the intervention group, ascorbic acid was stopped if anuric acute kidney injury occurred to avoid the accumulation of oxalates. Nephrotoxicity was assessed within one week of starting vancomycin therapy in both groups to avoid variation in vancomycin exposure.

### Demographic data

Patient demographics (age, gender, weight and height), comorbidities, concurrent medications, urine output, mean arterial pressure and vancomycin related factors (dose, route of administration and regimen) were recorded. Severity of illness was assessed using the APACHE II score (Acute Physiology and Chronic Health Evaluation II) and SOFA score (Sequential Organ Failure Assessment) for all patients within 24 hours of intensive care unit (ICU) admission.

### Clinical outcome measurements

Serum creatinine (S.cr) and blood urea nitrogen (BUN) samples were withdrawn on the starting day of vancomycin (baseline), day three, day seven and at additional times as needed. Creatinine Clearance (Cr.cl) was calculated using the Cockcroft-Gault equation utilizing ideal body weight if BMI was 18.5–24.9), adjusted body weight if BMI was >25 kg/m ^2^ or actual body weight in low body weight patients. ^26^

The primary outcomes of the study were the incidence of VAN and the absolute change in the creatinine parameters (serum creatinine and creatinine clearance) among the study groups. The guidelines defined VAN as an increase in serum creatinine of at least 0.5 mg/dL or ≥ 50% from baseline within seven days for at least two successive measurements from the starting time of the medication till 72 hours after its end. ^4^ A threshold increment of >0.3 mg/dL in S.cr over 48-hours was adopted by Kidney Disease: Improving Global Outcomes (KDIGO) and the Acute Kidney Injury Network (AKIN) as an indicator of VAN. Herein, VAN refers to the guideline-based definition of nephrotoxicity rather than KDIGO or AKIN criteria. VAN was staged according to the severity of AKI based on KDIGO 2012 guideline. ^27^ Mortality rate was the secondary outcome of the study.

### Serum concentration sampling

Blood samples were withdrawn to measure vancomycin serum trough level before the fourth dose (where steady state had been reached) by half an hour. ^4^ Blood samples were collected in heparinized blood tubes and were centrifuged immediately for four minutes at 1500 rpm, then the supernatants were separated and stored at -80°C. Plasma vancomycin trough levels were measured within four months using a liquid chromatography mass spectrometry (LC- MS/MS). A Shimadzu Prominence (Shimadzu Scientific Instruments, Columbia, MD, USA) series LC system equipped with degasser (DGU-20A3), solvent delivery unit (LC-20AB) with an auto-sampler (SIL-20 AC) was used to inject the aliquots of the samples on a Luna C18 (Phenomenex Inc., Torrance, CA, USA) 50 × 4.6 mm, 5 μm PS. The guard column was a Phenomenex C18 5 × 4.0 mm, 5 μm PS. All analyses were carried out at room temperature.

### Statistical analysis

A sample size of 40 patients across the two groups was estimated to be needed based upon the assumption that the vancomycin associated nephrotoxicity rate was 40% in the vancomycin (control) group ^6^ and 5% in the vancomycin-ascorbic acid (intervention) group, with 5% type I error and 20% type II error.

Statistics were done using the Statistical Package for Social Sciences ( SPSS) Version 25 (IBM SPSS Statistics, RRID:SCR_019096) (An open-access alternative that can perform an equivalent function is the R stats package (R Project for Statistical Computing, RRID:SCR_001905)). Continuous variables were reported as mean (SD) and were compared using Student’s t test as per non-significant Shapiro Wilk test ( *P-*value > 0.05), while non-normally distributed variables (as per significant Shapiro Wilk test ( *P-*value < 0.05) were compared using Mann Whitney U test. Categorical variables were compared using χ ^2^ test or Fisher’s exact test (when the assumptions of χ ^2^ were not fulfilled) and were reported as count (%).

The comparisons of the primary outcomes (serum creatinine, creatinine clearance and the absolute change in their values) were done using Student’s t-test as per continuous variables while the comparisons of the incidence of VAN (primary outcome) and the mortality rate (secondary outcome) were done using Fisher’s exact test and χ 2 test respectively, as per categorical variables. All data are available at Figshare. ^28^

## Results

**Figure 1.**
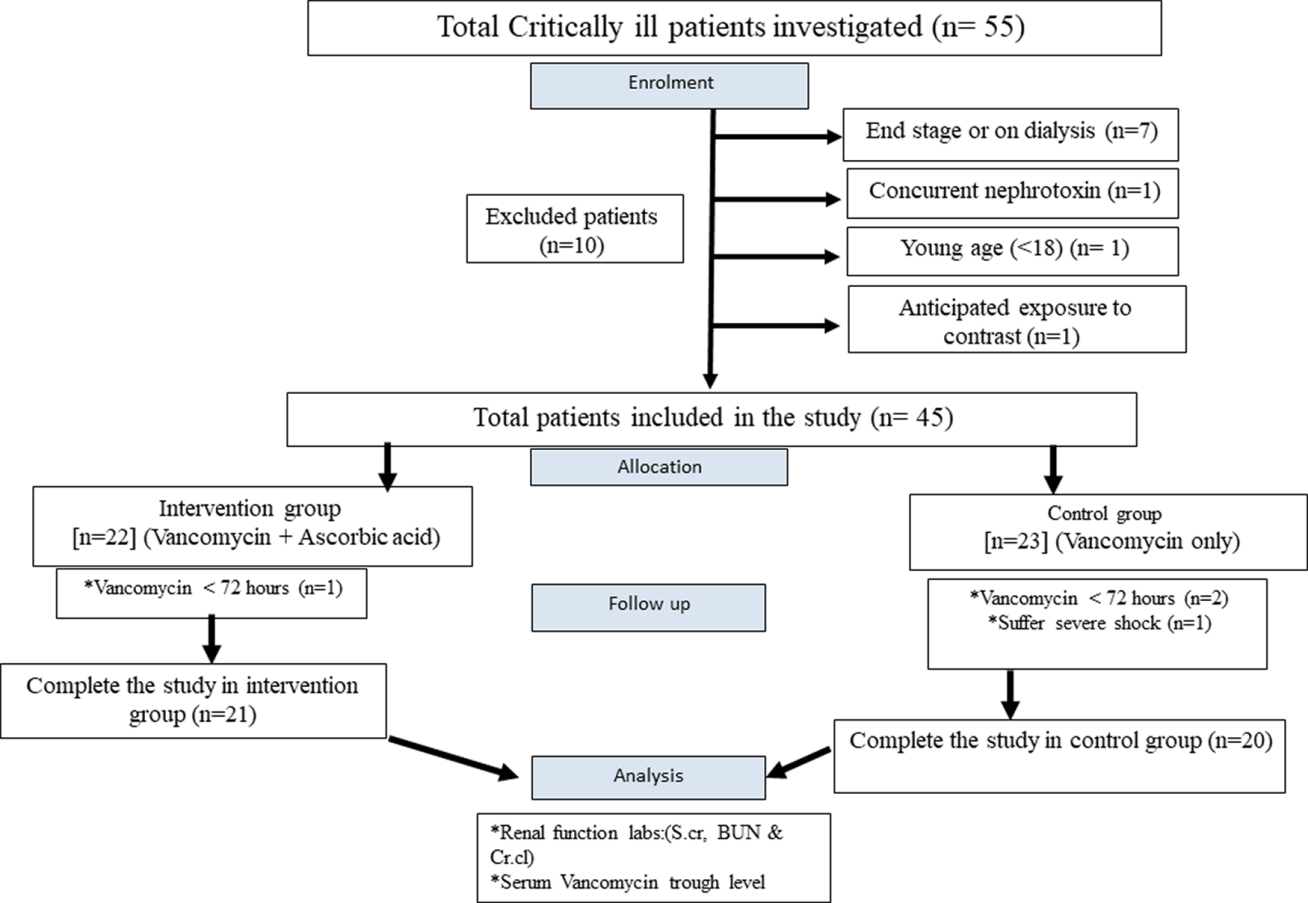
Detailed CONSORT flow diagram of the study. Abbreviations: S.cr, serum creatinine; BUN, blood urea nitrogen; Cr.cl, creatinine clearance.

Fifty-five critically ill patients with either confirmed or suspected MRSA infection were assessed for their eligibility for the study, however, only forty-one patients met both the inclusion and exclusion criteria and completed the study. They were randomly assigned to one of two groups as described in Figure 1.

Demographics, baseline clinical characteristics, vancomycin related factors and simultaneous potential nephrotoxins were comparable in both groups as shown in Table 1. The median APACHE II score and SOFA score also didn’t show any significant difference between the two groups. The main cause of infection in all patients was pneumonia (60.9%), and it was comparable in both groups. Other causes of infection are listed in Table 1, and they were all comparable in the two groups. Vancomycin treatment doses were higher in the intervention (ascorbic acid) group; however, they were non-statistically significant as described in Table 1. Total daily fluid intake, urine output, mean arterial pressure and the use of vasopressors were all comparable in both groups.

**Table 1.** Patients’ demographic data, underlying diseases and concurrent potential nephrotoxins in the study groups.

Parameters	Intervention group (n = 21)	Control group (n = 20)	*P-*value
Age, mean (SD) years	42.3 (16.5)	44.9 (15)	0.593 ^a^
Body weight, mean (SD) kg	74 (6.4)	72.7 (8.3)	0.580 ^a^
Male sex, no. (%)	17 (81)	11 (55)	0.074 ^b^
Mechanical ventilator, no. (%)	11 (52.4)	9 (45)	0.636 ^b^
APACHE II Score, median (IQR)	15 (10.5–16)	12 (9.25–16.75)	0.60 ^c^
SOFA score, median (IQR)	4.5 (3–5.25)	4.5 (3–6.25)	0.941 ^c^
Total fluid intake per day, mean (SD) mL	3501 (1275)	3060 (1241)	0.269 ^a^
Total fluid output per day, mean (SD) mL	2993 (809)	2905 (794)	0.725 ^a^
MAP, mean (SD) mmHg	89.9 (6.6)	89.6 (8)	0.884 ^a^
Underlying disease, no. (%) DM HTN Ischemic heart disease	3 (14.3) 5 (23.8) 2 (9.5)	4 (20) 7 (35) 3 (15)	0.697 ^d^ 0.431 ^b^ 0.663 ^d^
Chronic lung disease	1 (4.8)	3 (15)	0.343 ^d^
Stroke	1 (4.8)	3 (15)	0.343 ^d^
Concurrent Potential Nephrotoxins, no. (%)			
ACE inhibitors	4 (19)	2 (10)	0.663 ^d^
Furosemide IV/PO	1 (4.8)	4 (20)	0.184 ^d^
NSAIDs	4 (19)	4 (20)	1.000 ^d^
Piperacillin-Tazobactam	4 (19)	7 (35)	0.249 ^b^
Cephalosporin	3 (14.3)	4 (20)	0.697 ^d^
Vasopressors	2 (9.5)	1 (5)	1.000 ^d^
Main cause of infection, no. (%)			
Pneumonia HAP VAP	8 (38.1) 2 (9.5)	11 (55) 4 (20)	0.278 ^b^ 0.41 ^d^
Meningitis	3 (14.3)	1 (5)	0.606 ^d^
Infective endocarditis	3 (14.3)	2 (10)	1.00 ^d^
Skin and soft tissue	1 (4.8)	0 (0)	1.00 ^d^
Daily vancomycin dose per weight, mean (SD) mg/kg/day	35.2 (9.7)	30.5 (6.1)	0.07 ^a^
Trough level, mean (SD) mg/L	22.58 (14.35)	21.71 (14.72)	0.877 ^a^

Abbreviations: SD, standard deviation; IQR, interquartile range; MAP, mean arterial pressure; DM, diabetes mellitus; HTN, hypertension; ACE, angiotensin converting enzyme; IV, intravenous route; PO, per oral; NSAIDs, non-steroidal anti-inflammatory drugs; HAP, hospital associated pneumonia; VAP, ventilator associated pneumonia.

^a^ Based on Student’s t test.

^b^ Based on χ ^2^ test.

^c^ Based on Mann Whitney test.

^d^ Based on Fischer’s Exact test.

Serum creatinine, creatinine clearance (Cr.cl) means and blood urea nitrogen (BUN) median were all comparable at baseline in both groups. However, S.cr and Cr.cl showed a statistically significant difference between both groups when compared at the peak and the lowest values, respectively, Table 2. The mean absolute increase in S.cr concentration was significantly greater in the control group compared to the intervention group (difference of 0.29 mg/dL, 95% CI: 0.02 to 0.54, *P*-value = 0.036), Table 2. The average time for S.cr to peak was 5 and 4.2 days in the intervention and control group, respectively.

**Table 2.** Differences in laboratory investigations and mortality between the study groups.

Parameters	Intervention group (n = 21)	Control group (n = 20)	*P*-value
Serum creatinine (S.cr) (mean (SD) mg/dL)			
Baseline	0.73 (0.295)	0.75 (0.176)	0.768 ^a^
Peak	0.78 (0.27)	1.09 (0.56)	0.032 ^a^ ^*^
End	0.67 (0.21)	1.01 (0.61)	0.026 ^a^ ^*^
Creatinine clearance (Cr.cl) (mean (SD) mL/min)			
Baseline	126 (50.4)	107.9 (24.8)	0.148 ^a^	
Lowest	120.3 (47)	85.5 (30.1)	0.008 ^a^ ^*^	
End	132 (45.8)	93.1 (35.3)	0.004 ^a^ ^*^	
Blood urea nitrogen (BUN) (median (IQR) mg/dL)			
Baseline	21 (12.5–31.5)	17 (8.25–23.5)	0.240 ^b^	
Peak	25 (13–34)	21 (13.25–41.5)	0.927 ^b^	
End	13 (10–33)	19 (11.5–36)	0.566 ^b^	
Absolute difference in serum creatinine (peak baseline) (mean (SD) mg/dL)	0.05 (0.12)	0.34 (0.55)	0.036 ^a^ ^*^	
Absolute difference in creatinine clearance (lowest baseline) (mean (SD) ml/min)	−5.9 (17.8)	−22.3 (30.4)	0.04 ^a^ ^*^	
Incidence of acute kidney injury (AKI), no. (%)	1 (4.7)	5 (25)	0.093 ^d^	
Mortality within 28 days, no. (%)	4 (19)	8 (40)	0.141 ^c^

Abbreviations: SD, standard deviation; IQR, interquartile range.

^a^ Based on Student’s t test

^b^ Based on Mann-Whitney U test

^c^ Based on χ _2_ test

^d^ Based on Fisher’s exact test

* Statistically significant ( *P*-value <0.05)

-Baseline S.cr: S.cr level on the first day of study or the day before it.

-Peak S.cr: highest level of S.cr from the starting day of vancomycin medication and till its end.

-End S.cr: value of the s.cr at the end of the vancomycin treatment.

-Baseline Cr.cl: Cr.cl level on the first day of study or the day before using the baseline S.cr.

-lowest Cr.cl: is the lowest value of Cr.cl that is corresponding to the peak S.cr.

-End Cr.cl: value of the Cr.cl at the end of the vancomycin treatment.

In a similar manner, the mean absolute decline in Cr.cl concentration was significantly greater in the control group compared to the intervention group (difference of 16.4 mL/min, 95% CI: −32.12 to −0.79, *P*-value = 0.04), Table 2. Despite the significant increase observed in S.cr, BUN peak concentrations were not significantly different in either group, Table 2.

**Figure 2.**
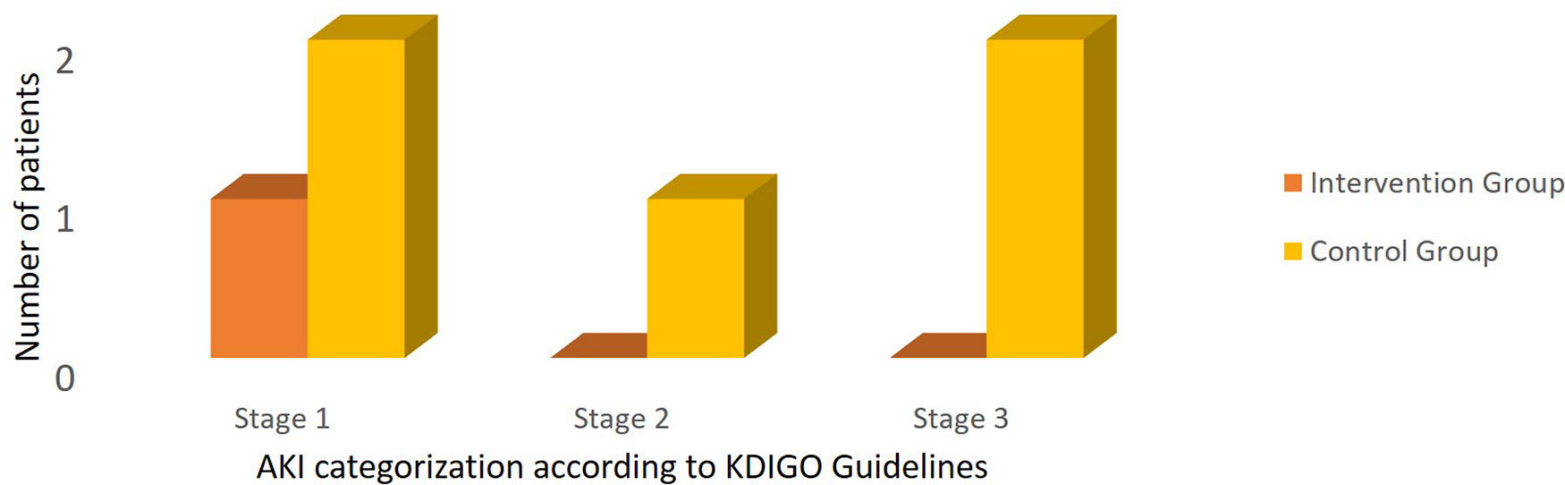
Acute kidney injury categorization.

According to the definition of VAN, acute kidney injury occurred in 6 of 41 patients (14.6%) – 1 of 21 patients (4.7%) in the intervention group and 5 of 20 patients (25%) in the control group (RR: 0.19, CI: 0.024–1.49, *P*-value = 0.093). By applying the new definition of VAN that is adopted by AKIN and KDIGO, the number of patients suffering VAN didn’t change in either group. AKI categorization is summarized in Figure 2. No adverse effects related to ascorbic acid were detected in the intervention group. The ICU mortality rate within 28 days was higher in the control group compared to the intervention group, however, it didn’t reach a statistically significant level, Table 2.

## Discussion

Vancomycin associated nephrotoxicity (VAN) in critically ill patients is a topic of debate. Many factors may contribute to increasing VAN incidence in this population such as being infected with more resistant pathogens that require larger doses of treatment, alterations in the volume of distribution and concurrent administration of potential nephrotoxic medications. ^29, 30^

In the current study, the incidence of VAN was 25% in the control group. A comparable incidence of 29.5% was reported in a previous study that was performed on post cardiac surgery patients treated with vancomycin. ^31^ Also, an incidence rate of 27.2% was obtained from a study performed on pediatric critically ill patients. ^32^

Several experimental studies have investigated the possible nephroprotective role of many antioxidants and they showed promising results in attenuating proximal renal tubular injury, especially upon ascorbic acid administration. ^21, 33^ Therefore, the aim of the current study was to investigate the potential nephro-protective role of ascorbic acid against VAN in critically ill patients.

Ascorbic acid is a potent antioxidant capable of scavenging a wide range of reactive oxygen species and prevents their damaging effect to macromolecules such as lipids, DNA, and proteins. It has been reported to have the lowest toxicity of all vitamins. ^22^ It is a known precursor of oxalate that may lead to renal failure secondary to hyperoxaluria, However, acute oxalate nephropathy had been reported to occur after the administration of a single dose of 2.5 grams of intravenous ascorbic acid in patients with previous renal injury. ^22^ Therefore, 4 grams of ascorbic acid per day (divided in two doses) was selected as this dose has been shown to be effective against contrast induced nephrotoxicity (CIN) ^25^ and was reported to be safe without increasing the risk of urinary oxalate. ^30^

In the present study, the absolute difference in S.cr and Cr.cl levels were significantly lower in the ascorbic acid group in comparison with the control group. These findings were consistent with what was obtained from rats and mice models. ^21, 33^ The average time for the S.cr to inflate was five and four days in the intervention and control group, respectively, and this is similar to the range reported by Van Hal *et al* in their meta-analysis. ^14^

Regarding the incidence of VAN, co-administration of ascorbic acid with vancomycin reduced the absolute risk of VAN incidence by 20.3%, however, this reduction didn’t reach a statistically significant level (RR: 0.19; CI: 0.024–1.49; *P*-value = 0.093). Nevertheless, a 20.3% reduction in the absolute risk of VAN and an approximate 80% reduction in its relative risk should be considered clinically. Mortality was higher in the control group (40%), but it didn’t show a significant difference when compared to the study group (19%). It has been reported in several studies that the mortality rate due to VAN is between 15–60%, and it varies according to the degree of renal injury that has occurred and the studied population. ^2^

Several reasons may have contributed to the lack of a statistically significant reduction in the proportion of patients developing VAN. Firstly, the pilot nature of the study may have been insufficient to detect the reduction in VAN incidence statistically. Therefore, further randomized trials with a larger sample size are recommended. Secondly, because of the lack of ascorbic acid as an intravenous injection dosage form in Egypt at the study time, it was administered orally to patients rather than intravenously. The bioavailability of oral dosage forms of ascorbic acid is 36% for the two-gram dose, which is much lower than the intravenous one. ^34^ This might have affected the expected nephro-protective effect of the antioxidant. Thirdly, although vancomycin doses were statistically comparable in both groups, it was noticed that the mean vancomycin daily dose per body weight was higher in the ascorbic acid group compared to the control group. This can be attributed to the higher number of patients suffering from meningitis and infective endocarditis in the intervention group. Consequently, these higher doses might have contributed in increasing the incidence of VAN in the intervention group. ^35, 36^ Other potential confounding influences such as hypotension and the need for vasopressors during the study were minimal and comparable among both groups.

## Conclusion

This preliminary study revealed that ascorbic acid co-administration with vancomycin reduces the absolute increase in S.cr concentration and the absolute decrease in Cr.cl resulting from vancomycin administration. It also reduced the absolute risk of VAN in critically ill patients by 20.3%, however, it didn’t reach a statistically significant level. Further large multicenter prospective randomized clinical trials are recommended to confirm the efficacy of ascorbic acid as a nephro-protectant against VAN.

## Data availability

### Underlying data

Figshare: Underlying data for ‘Impact of ascorbic acid in reducing the incidence of vancomycin associated nephrotoxicity in critically ill patients: A preliminary randomized controlled trial’, https://doi.org/10.6084/m9.figshare.15710004.v1. ^28^

This project contains the following underlying data:

• Data file 1: Demographic data, underlying diseases, concurrent medications and laboratory data in the study groups.
• Original trial protocol.

### Reporting guidelines

Figshare: CONSORT checklist for ‘Impact of ascorbic acid in reducing the incidence of vancomycin associated nephrotoxicity in critically ill patients: A preliminary randomized controlled trial’, https://doi.org/10.6084/m9.figshare.15710004.v1. ^28^

Data are available under the terms of Creative Commons Zero “No rights reserved” data waiver (CC0 1.0 Public domain dedication).

## Consent

Written informed consent was obtained from all individual participants included in the study.
