# Supplementary material for: Impact of ascorbic acid in reducing the incidence of vancomycin associated nephrotoxicity in critically ill patients: A preliminary randomized controlled trial
Source: F1000Res. 2021 Sep 16;10:929. [Version 1] doi: 10.12688/f1000research.55619.1 (PMC8479849; doi:10.12688/f1000research.55619.1)
Supplement: Peer review history

# Peer review: Reviewer response for version 1

**DOI:** 10.5256/f1000research.59212.r94495

**Published:** 2021-09-28

**Authors:** Mona F. Schaalan

**Affiliations:** 1 Pharmacy Practice and Clinical Pharmacy Department, Translational and Clinical Research Unit, Faculty of Pharmacy, Misr International University (MIU), Cairo, Egypt

First, I appreciate this opportunity to review this interesting article " Impact of ascorbic acid in reducing the incidence of vancomycin associated nephrotoxicity in critically ill patients: A preliminary randomized controlled trial."

 This study assessed the nephro-protective effect of oral ascorbic acid against VAN in critically ill patients. The current study is a prospective randomized open-label trial. Generally, the study is appropriately designed with sufficient sample size. The results revealed that concomitant administration of oral ascorbic acid with vancomycin preserved renal function and reduced the absolute risk of VAN by 20.3%, yet did not reach statistical significance. This is appropriately discussed and rationalized. The authors also addressed their limitations of the study at the end of the discussion.

 However, there are some minor comments that need to be clarified and added to the manuscript.

1. The authors used 2g ascorbic acid twice daily half an hour before the vancomycin dose for the patients assigned to the intervention group. ^25^ Reading the reference no. 25, they used a different dose. The authors should discuss the difference in the dosing used. The authors should also discuss how the dosage used, 2g twice daily would not put the patients at risk of oxalate accumulation.
2. The presentation of results in Table 2 needs to be better illustrated in figures and graphs to track the changes in values; e.g. a graph showing the incidence of VAN and change of S.cr and Cr.cl over time in the control and treated groups would help the reader observe the extent of change.
3. Figure 2. is not sufficiently described and needs the histogram to be more clear in terms of units in the y axis and the magnitude of bars in both arms.
4. In the discussion section, 4th paragraph, third line; however, should be written small initial and preceded by semi colon. "It is a known precursor of oxalate that may lead to renal failure secondary to hyperoxaluria, However, acute oxalate nephropathy …." 

To this end, my decision is approval of the manuscript.

Is the work clearly and accurately presented and does it cite the current literature?

Yes

If applicable, is the statistical analysis and its interpretation appropriate?

Yes

Are all the source data underlying the results available to ensure full reproducibility?

Yes

Is the study design appropriate and is the work technically sound?

Yes

Are the conclusions drawn adequately supported by the results?

Yes

Are sufficient details of methods and analysis provided to allow replication by others?

Yes

Reviewer Expertise:

Clinical Pharmacology in the areas of diabetes, hypertension, and Metabolic Syndrome, and Kidney diseases.

I confirm that I have read this submission and believe that I have an appropriate level of expertise to confirm that it is of an acceptable scientific standard.

# Peer review: Reviewer response for version 1

**DOI:** 10.5256/f1000research.59212.r94499

**Published:** 2021-09-21

**Authors:** Abdelrahman Fouda

**Affiliations:** 1 University of Arkansas for Medical Sciences, Little Rock, AR, USA

The article by Dr. El-Sherazy et al. examined the effect of concomitant administration of ascorbic acid to reduce the incidence of vancomycin-associated nephrotoxicity in critically ill patients. The study is a prospective randomized open-label trial. The study is well designed and the sample size is adequate. The authors found that co-administration of ascorbic acid with vancomycin preserved renal function and reduced the absolute risk of VAN by 20.3%, however, the reduction in VAN incidence was not statistically significant. There were some limitations in the study which the authors addressed in the discussion section such as using ascorbic acid as an oral dose instead of intravenous injection due to lack of availability.

 My comment is that the study would benefit from representing some of the key data as figures in addition to the numbers in tables at least for the primary outcomes. For example, a graph showing the incidence of VAN and change of S.cr and Cr.cl over time in the control and intervention groups would give the reader an idea of the change in these parameters with and without ascorbic acid administration. In addition, graphical representation of the numbers of absolute increase in S.cr and decrease in Cr.cl would help the reader appreciate the magnitude of change.

Is the work clearly and accurately presented and does it cite the current literature?

Yes

If applicable, is the statistical analysis and its interpretation appropriate?

Yes

Are all the source data underlying the results available to ensure full reproducibility?

Yes

Is the study design appropriate and is the work technically sound?

Yes

Are the conclusions drawn adequately supported by the results?

Yes

Are sufficient details of methods and analysis provided to allow replication by others?

Yes

Reviewer Expertise:

Translational research

I confirm that I have read this submission and believe that I have an appropriate level of expertise to confirm that it is of an acceptable scientific standard.
